# A Medical Globe-Trotter

**Published:** 1900-02-17

**Authors:** 


### A MEDICAL GLOBE-TROTTER.

Dr. Abram Brothers is an American, who, although he came over to Europe for rest, found time to get in a large amount of hospital sight-seeing among the gynæcologists in Berlin, Paris, and London. Returning now to his native soil he records his experiences for the benefit of the readers of the New York *Medical Record*. Without doubt he saw a good deal, and he tells his tale with that easy lack of reticence about personal peculiarities which is dear to the heart of the American newspaper reader.

Clearly some of the Berlin gynæcologists have a keen eye to business, and do not care to make a present of their experience to medical globe-trotters. In order to be admitted to the operating-rooms, he says it is customary in Berlin for the visitor first to call on the operator and present his card. But although he found the said operators very cordial during one or two visits, he also found that after the first one or two it was made "painfully clear that, in the absence of 'taking the course,' such visits would be cheerfully excused." One morning he visited at the large public Kaiserliche Krankenhaus, the surgical clinic of "a young surgeon named Lexer," in company with two American colleagues, when the surgeon straightways sent up the janitor to see if they had taken tickets for the course So that although, as is stated, much good work in all branches of medicine and surgery can be done in Berlin, it is regretfully added that not only is the work scattered, but that it is "quite expensive."

In regard to London, the following is the summing up of his experience: "Of course, in the short space of nine days one can hardly begin to form a fair conception of the quality of work done in a large city like London. But of one thing I am certain, and that is that there is less pure aseptic surgery done in London than in Berlin, Paris, or New York. . . . I have seen the nurse in charge of sponges during an abdominal hysterectomy push tables, hand over basins, and otherwise subject herself to infection, without washing her hands a single time. I have seen operators of surpassing skill wipe the nose, scratch the forehead, twirl the moustache, or adjust eyeglasses, and proceed with the same hand in their work (sometimes in the abdominal cavity) without dipping it even in water."

